# Evaluation of the impacted upper canine and maxillary sinus dimensions using cone beam computed tomography

**DOI:** 10.1590/1807-3107bor-2026.vol40.031

**Published:** 2026-06-12

**Authors:** Merve KURNAZ, Emine KAYGISIZ, Gülsün AKAY, Kahraman GÜNGÖR, Tuba TORTOP

**Affiliations:** (a) Istanbul Kent University, School of Dentistry, Department of Orthodontics, Istanbul, Turkey.; (b) Private Practice, Bursa, Turkey.; (c) Gazi University, School of Dentistry, Department of Dentomaxillofacial Radiology, Ankara, Turkey.; (d) Gazi University, School of Dentistry, Department of Orthodontics, Ankara, Turkey.

**Keywords:** Maxillary sinus, Cone-Beam Computed Tomography

## Abstract

This study aimed use cone beam computed tomography (CBCT) to compare the maxillary sinus volume and transverse dimensions between sides with and without palatally impacted maxillary canines. The CBCT records of 47 patients with unilateral palatally impacted canines (18 males, 29 females; mean age: 22.19 ± 7.71 years) were included. The maxillary sinus volume was measured using semiautomatic segmentation, and transversal maxillary dimensions were assessed in the posterior region. Deviation of the nasal septum and alveolar pneumatization were also evaluated. Statistical analyses were performed using SPSS 25.0 with a significance level set at p < 0.05. As compared with the nonimpacted side, the maxillary and molar basal widths were significantly smaller and the septum deviation angle was greater on the impacted side (p < 0.05). A one-unit increase in the septal deviation angle increased the probability of impaction by 1.177 times. A negative correlation was detected between the maxillary sinus intensity and zygomatic width, molar basal width, and molar alveolar width (r = 0.431, 0.316, and 0.351, respectively). The incidence of pathological sinus findings did not differ significantly between sides (38.3% vs. 51.06%). Similarly, the rates of alveolar pneumatization were comparable (51.06% vs. 48.94%). The mean sinus volumes were 13,147.96 voxels on the impacted side and 13,194.38 voxels on the nonimpacted side, with no significant difference (p > 0.05). In conclusion, although palatally impacted maxillary canines are associated with narrower posterior maxillary transverse dimensions and increased septal deviation, they do not have a considerable effect on the maxillary sinus volume. These findings provide clinically relevant information for orthodontic diagnosis and surgical planning.

## Introduction

Maxillary canines exhibit the second highest prevalence of impaction, occurring twice as frequently in women than in men.^1^ The prevalence of palatally impacted canines is two to three times higher than that of buccally impacted canines.^1-3^

Tooth eruption entails the axial transition of a tooth from its initial nonfunctional position within the alveolar bone to functional occlusion.^4^ Eruption is a physiological process that plays a crucial role in the proper development of the alveolar bone. Nevertheless, tooth impaction can impede the local development of the alveolar bone and adjacent structures.^5^

Cone beam computed tomography (CBCT) helps determine the direction and surgical approach for impacted tooth extraction.^6-8^ In addition, to determine the suitable regions for mini screws, which are frequently used for the eruption of impacted canine teeth with orthodontic forces, it is necessary to evaluate the maxillary bone and arch dimensions.^9,10^

In studies in which impacted canines are evaluated with CBCT, millimetric measurements are frequently obtained on the maxillary bone.^6-8^ Tadinada et al.^6^ retrospectively evaluated the arch perimeter, buccopalatal width, and alveolar height in unilateral palatally impacted canines via CBCT and reported that these values were significantly lower on the side of the impacted tooth. By examining the maxillary width at four levels, Arboleda-Ariza et al.^7^ found that patients with unilateral or bilateral impacted maxillary canines exhibited reduced maxillary transverse dimensions compared with those without impacted teeth. In another study that focused on unilaterally impacted canines with CBCT images, the authors measured skeletal and dentoalveolar variables related to the height and width of the maxilla using cross-sectional images. The distance from the median raphe to the first bicuspid was narrower on the side where the impacted tooth was located.^8^

Volume measurement has become achievable through software that enables modeling and segmentation via semiautomatic techniques involving computed tomography (CT) and magnetic resonance images. These techniques are compatible with three-dimensional (3D) imaging methods and facilitate morphometric measurements. The software enables the segmentation of structures based on Hounsfield units. Moreover, it is possible to differentiate anatomically complex structures from surrounding ones, generate a 3D model, measure the area and volume of the isolated structure, and compare tissue densities.^11^ CBCT is extensively used as the “gold standard” for imaging the paranasal sinuses.^12^ In addition, CBCT has acceptable sensitivity, specificity, and diagnostic accuracy for determining alveolar pneumatization and the relationship between the tooth root and sinus.^13^

In light of this information, developmental differences can be observed in the bone and tissues that surround an impacted canine tooth. However, only a limited number of studies^6-10^ have been conducted on this topic, using only dimensional measurements. Therefore, the primary aim of the current research was to compare the volume of the maxillary sinus and the dimensions of this region between the sides with and without upper impacted canines using semiautomatic segmentation and the development of 3D imaging technology in the same patients. Our second aim was to evaluate alveolar pneumatization of the maxillary sinus according to the impacted canine tooth. Our null hypothesis stated that there is no difference in sinus volume or dimensions between the impacted and nonimpacted sides.

## Methods

### Study design

We conducted this retrospective observational study in accordance with the Strengthening the Reporting of Observational Studies in Epidemiology (STROBE) guidelines.^14^ The Ethics Committee of Gazi University (approval No: #06.06.2023-E.673254) approved the study protocol.

### Study setting and data collection

We conducted the study at the Department of Oral and Maxillofacial Radiology, Gazi University. We retrospectively reviewed the institutional CBCT image archive between January 2014 and December 2022. All CBCT images were acquired using the same radiographic unit (Planmeca Promax 3D Mid, Planmeca, Helsinki, Finland) and identical exposure parameters (90 kVp, 9–12 mA, exposure time 13.2 seconds, voxel size 0.4 mm). All participants provided informed consent at the time of imaging.

### Participants

We screened a total of approximately 6,000 CBCT scans. Participants were selected according to the following eligibility criteria.

- Inclusion criteria: a) unilateral palatally impacted maxillary canine; b) CBCT scans displaying the impacted tooth and the maxillary sinus area; c) age >13 years; d) absence of other supernumerary, missing, or impacted teeth except for the third molars; and e) high-quality CBCT images without artifacts.
- Exclusion criteria: patients with congenital tooth agenesis, cleft lip and/or palate, sinus pathology, history of orthodontic treatment, craniofacial syndromes, or any previous surgical intervention in the maxillofacial region.

A total of 47 patients (18 males, 29 females; mean age = 22.19 ± 7.71 years) met the inclusion criteria and were included in the study.

### Variables and definitions

The main outcome variable was maxillary sinus morphology, which was evaluated through volumetric and linear measurements. Predictor variables included impaction side (right/left) and the presence of alveolar pneumatization. The pathological sinus findings were defined and scored (0–4) according to Pazera et al.^13^ based on Schneiderian membrane thickening.

### Data sources and measurement procedures

**Figure 1:**
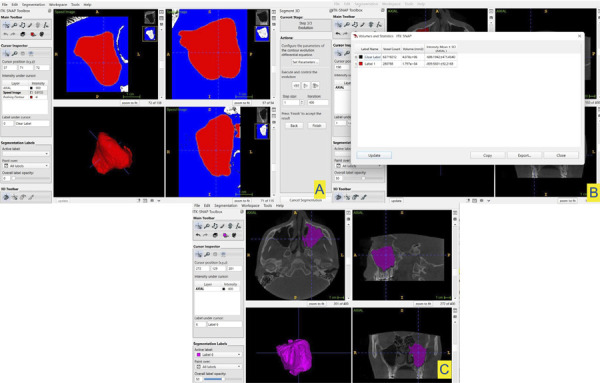
The volume was calculated via semiautomatic segmentation of the maxillary sinus using the ITK-SNAP 3.8.0 (Cognitica Philadelphia, USA) software program.

We imported the CBCT data into ITK-SNAP 3.8.0 software (Cognitica, Philadelphia, USA). The maxillary sinus volume was determined using semiautomatic segmentation (Figure 1).

(A) Segmentation and three-dimensional reconstruction of the maxillary sinus. (B) Display screen illustrating the visualization of sinus volume and density. The complete three-dimensional visualization of the maxillary sinus across the entire CBCT dataset.)

**Figure 2:**
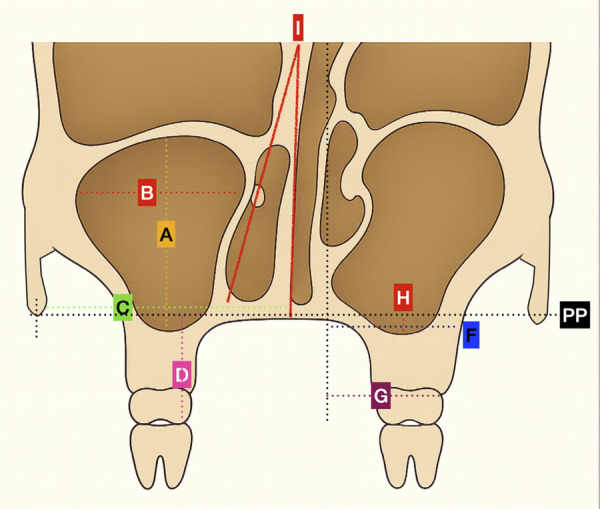
Linear and angular measurements used in this study.

Linear and angular measurements were made on the coronal sections of each CBCT scan (Figure 2). A single calibrated investigator (M.K.). obtained all measurements. To assess intraobserver reliability, one-third of the images were randomly remeasured after 15 days, and the intraclass correlation coefficients were calculated.

(A) Maxillary sinus height: vertical distance between the highest and lowest points of the sinus at the level of the maxillary first molars. (B) Maxillary sinus width: horizontal distance between the most lateral points of the sinus at the same level. (C) Zygomatic width: distance from the lowest point of the zygomatic bone to the nasal midline. (D) Maxillary height: distance from the palatal tubercle of the first molar to the palatal plane. (E) Maxillary width: distance from the deepest point of the alveolar process to the nasal midline. (F) Molar basal width: distance from the deepest point of the alveolar process to the midline of the nasal concha. (G) Molar alveolar width: distance from the gingival attachment of the maxillary first molar to the midline of the sinus. (H) Maxillary sinus basal depth: distance between the deepest point of the sinus and the palatal plane. (I) Septum deviation angle: angle between the nasal midline plane and the plane passing through the maxillary first molars.

### Bias control

All scans were obtained using the same CBCT unit and standardized exposure settings to minimize measurement bias. Measurements were performed under identical lighting conditions. We verified repeatability using ICC testing; one-third of the measurements were reassessed after 15 days, and justification for this procedure was provided. However, because the examiner was aware of the impacted side during the measurements, this could be considered a limitation of the study.

### Study size and power

We calculated the sample size using G*Power 3.1.9.7 software (Franz Faul, Kiel University, Germany). A post hoc power analysis indicated that with an effect size (d) of 0.53, an alpha error probability (α) of 0.05, and two groups of 47 participants each, the achieved statistical power (1 – β) was 0.8179 (81.79%), which confirms that the sample size was sufficient for the study.

### Quantitative variables and statistical analysis

We performed statistical analyses using SPSS version 25.0. We assessed the normality of the variables through histogram plots and the Kolmogorov–Smirnov test. Descriptive statistics were presented using mean, standard deviation, median, and minimum–maximum values. The categorical variables were compared using the chi-square test. For comparisons between the two groups, we used the Mann–Whitney U test for nonnormally distributed (nonparametric) variables and the independent samples t- est for normally distributed (parametric) variables. Spearman’s correlation test was used to analyze the relationships among measurement variables. We calculated the intraclass correlation coefficients to assess the agreement between repeated measurements. A p-value of 0.05 was considered statistically significant.

## Results

The repeatability of the impacted and nonimpacted sides’ intraclass correlation coefficients was close to 1.000, except for the maxillary sinus intensity and molar basal width on the impacted side (0.744 and 0.795, respectively).

Of the patients, 61.7% were female and 38.3% were male. The average age of the participants was 22.19 ± 7.71 years.

Table 1 presents the mean values, standard deviations, and comparative statistical values of the measurements on the side with and without impaction. The maxillary width and molar basal width values were significantly lower on the impacted side, whereas the septum deviation angle was greater (p < 0.05). A one-unit increase in the deviation angle of the septum resulted in a 1.177-unit increase in the likelihood of being impacted (Table 1).

**Table 1:** Comparative statistical evaluation of linear, angular, and volumetric parameters between the impacted and non-impacted canine sides.

Variables	Impacted side		Non-impacted side		p^1^	p^2^	p^3^	Exp(B)
	X±Sd	Median	X±Sd	Median				
		(Min,Max)		(Min,Max)				
Sinus volume	13147.96±4729.78	12880	13194.38 ± 4780.1	12880	0.928			
		-4692,3072		-5665,257				
Sinus intensity	-515.82±575.27	-763.15	-528.98 ± 575.56	-767.11		0.912		
		(-870.64,882.03)		(-864.88,863.09)				
Maxillary sinus height (A)	29.27±6.09	28.76	29.46 ± 5.73	29.21	0.985			
		(13.93,47.04)		(19.85,43.26)				
Maxillary sinus width (B)	22.7±4.26	22.51	22.45 ± 3.34	22.57	0.783			
		(13.87,34.43)		(10.94,28.4)				
Maxillary sinus basal depth (H)	3.75±2.97	3.88	4.5 ± 2.18	4.14		0.167		
		(-11.7,10.05)		(-1.62,8.95)				
Maxillary height (D)	19.82±3.35	19.11	19.43 ± 2.69	19.21	0.797			
		(15.04,33.68)		(15.25,25.95)				
Maxillary width (E)	28.14±3.04	28.35	29.5 ± 2.49	29.48	0.010*		0.553	1.048 (0.897,1.224)
		(16.34,36.05)		(22.64,34.89)				
Zygomatic width(C)	47.32±3.42	47.39	47.32 ± 4.83	48.2	0.578			
		(38.89,54)		(30.08,56.31)				
Molar basal width (F)	20.45±1.96	20.51	21.53 ± 3.16	21.47	0.030*		0.056	0.849 (0.718,1.004)
		(16.47,24.32)		(13.8,31.19)				
Molar alveolar width (G)	18.28±2.01	18.3	19.15 ± 3.2	19.48	0.131			
		(13.7,22.31)		(10.06,30.39)				
Septum deviation angle (I)	20.15±3.79	20.2	18.36 ± 2.9	18.1	0.013*		0.016*	1.177 (1.031, 1.345)
		(10.3,30.59)		(11.9,25.14)				

Values are expressed as mean ± standard deviation (X ± Sd) and median (minimum–maximum). p₁: Mann–Whitney U test; p₂: Independent t-test; p₃: Binary logistic regression analysis. Exp(B) indicates the odds ratio derived from the logistic regression model. *p <0.05 as statistically significant.

Table 2 shows the correlations of the parameters on the impacted canine side. There was no correlation between the parameters. Table 3 gives the correlations of the parameters on the nonimpacted canine side. The maxillary sinus intensity was inversely correlated with zygomatic width, molar basal width, and molar alveolar width (r = −0.431, −0.316, and 0.351, respectively).

**Table 2:** Correlation of parameters on impacted canine side.

Variables		Zygomatic width (C)	Maxillary height (D)	Maxillary width (E)	Molar basal width (F)	Molar alveolar width (G)
Maxillary sinus height (A)	r	0.140	-0.171	-0.036	-0.266	-0.175
	p	0.349	0.249	0.811	0.070	0.239
Maxillary sinus width (B)	r	-0.225	-0.121	0.200	0.109	-0.012
	p	0.128	0.416	0.178	0.464	0.938
Maxillary sinus basal depth (H)	r	-0.021	0.046	0.228	0.047	-0.024
	p	0.889	0.760	0.122	0.754	0.875
Sinus volume	r	0.064	-0.143	0.193	-0.004	-0.268
	p	0.671	0.339	0.193	0.977	0.068
Sinus intensity	r	-0.282	0.164	0.272	0.105	0.157
	p	0.055	0.272	0.064	0.483	0.292
Septum deviation angle (I)	r	0.053	-0.003	0.141	-0.143	-0.005
	p	0.723	0.983	0.343	0.339	0.974

A. Maxillary sinus height: the distance between the two farthest points vertically within the maxillary sinus at the level of the maxillary first molar teeth. B. Maxillary sinus width: the distance between the two farthest points horizontally within the maxillary sinus at the level of the maxillary first molar teeth. C. Zygomatic width: the distance between the lowest point of the zygomatic bone and the midline of the nasal bone. D. Maxillary height: the distance between the palatal tubercle of the maxillary first molar teeth and the palatal plane. E. Maxillary width: the distance between the deepest point of the alveolar process at the level of the maxillary first molar teeth and the midline of the nasal sinus. F. Molar basal width: the distance between the deepest point of the alveolar process at the level of the maxillary first molar teeth and the midline of the concha. G. Molar alveolar width: the distance between the gingival attachment level of the maxillary first molar teeth and the midline of the maxillary sinus. H. Maxillary sinus basal depth: the distance between the deepest point of the sinus and the palatal plane. I. Septum deviation angle: the angle formed between the plane of the midline of the nasal bone and the plane formed by the maxillary first molar. *p < 0.05 as statistically significant. Spearman’s correlation test was employed.

**Table 3:** Correlation of parameters on the non-impacted canine side.

Variable		Zygomatic width (C)	Maxillary height (D)	Maxillary width (E)	Molar basal width (F)	Molar alveolar width (G)
Maxillary sinus height (A)	r	-0.027	-0.172	0.053	0.060	0.093
	p	0.857	0.247	0.722	0.691	0.536
Maxillary sinus width (B)	r	0.030	-0.158	0.277	0.088	0.168
	p	0.842	0.288	0.059	0.556	0.260
Maxillary sinus basal depth (H)	r	-0.130	-0.136	0.142	0.144	0.175
	p	0.384	0.363	0.342	0.335	0.240
Sinus volume	r	0.191	0.030	0.236	0.183	-0.009
	p	0.198	0.840	0.110	0.219	0.952
Sinus intensity	r	-0.431*	0.090	0.845	-0.316*	-0.351
	p	0.002	0.546	0.236	0.030	0.016
Septum deviation angle (I)	r	-0.134	0.265	0.172	0.037	-0.027
	p	0.368	0.072	0.247	0.804	0.855

A. Maxillary sinus height: the distance between the two farthest points vertically within the maxillary sinus at the level of the maxillary first molar teeth. B. Maxillary sinus width: the distance between the two farthest points horizontally within the maxillary sinus at the level of the maxillary first molar teeth. C. Zygomatic width: the distance between the lowest point of the zygomatic bone and the midline of the nasal bone. D. Maxillary height: the distance between the palatal tubercle of the maxillary first molar teeth and the palatal plane. E. Maxillary width: the distance between the deepest point of the alveolar process at the level of the maxillary first molar teeth and the midline of the nasal sinus. F. Molar basal width: the distance between the deepest point of the alveolar process at the level of the maxillary first molar teeth and the midline of the concha. G. Molar alveolar width: the distance between the gingival attachment level of the maxillary first molar teeth and the midline of the maxillary sinus. H. Maxillary sinus basal depth: the distance between the deepest point of the sinus and the palatal plane. I. Septum deviation angle: the angle formed between the plane of the midline of the nasal bone and the plane formed by the maxillary first molar. *p < 0.05 as statistically significant. Spearman’s correlation test was employed.

The incidence of pathological sinus findings was 38.30% for scores of 1 and 2 on the impacted side and 51.06% for scores of 1 on the nonimpacted side. The percentage of pneumatization was 51.06% on the impacted side and 48.94% on the nonimpacted side. The comparisons of pathological sinus findings and pneumatization between the impacted and nonimpacted sides revealed no significant differences (Table 4).

**Table 4:** Percentages of pneumatization and pathological sinus findings comparing impacted vs. non-impacted sides .

Variable		Non-impacted side		Impacted canin side		p-value
		n	%	n	%	
Pathological sinus findings	0: None	0	%0.00	0	%0.00	0.625
	1: Mucosal thickening	24	(51.06)	18	(38.30)	
	2: Polypoidal thickening	13	(27.66)	18	(38.30)	
	3: Partial opacity	5	(10.64)	6	(12.77)	
	4: Total opacity	5	(10.64)	5	(10.64)	
Pneumatization	-	24	(51.06)	23	(48.94)	0.837
	+	23	(48.94)	24	(51.06)	

The Chi-Square test. *p < 0.05 as statistically significant.

## Discussion

The effect of impacted or missing teeth on the surrounding structures such as the maxillary sinus and nasal cavity has long been a subject of clinical interest, particularly for diagnostic assessment and treatment planning.^15,16^Due to its high accuracy and low radiation exposure, CBCT is recognized as the gold standard for paranasal sinus evaluation.^1 2^ In the present study, we performed a detailed assessment of unilateral palatally impacted canine cases to compare the maxillary sinus volume and transverse maxillary dimensions using semiautomatic 3D segmentation (ITK-SNAP 3.8.0 [Cognitica, Philadelphia, USA]) for the first time in this context. In addition, we evaluated alveolar pneumatization associated with impacted canines.

Although prior CBCT studies investigated the morphological effects of impacted canines, only a limited number focused specifically on maxillary sinus volume.^6-9,16^ Tassoker et al.^17^ examined sinus volumes in patients with impacted teeth but included various tooth types. Horáček et al.^18^ evaluated the effects of treatment on sinus volume, whereas Oz et al.^16^ found smaller sinus volumes on the impacted side, although the difference was not significant. Similarly, we found no significant volumetric difference in our study, supporting the null hypothesis that palatally impacted upper canines do not affect sinus volume. Nevertheless, the impacted side consistently exhibited slightly smaller values, in accordance with the findings of Tassoker et al.^17^

The present split-mouth design minimized interindividual variability and allowed direct comparison within the same subject, ensuring methodological consistency. Tadinada et al.^6^ and Arboleda-Ariza et al.^8^ adopted comparable approaches to strengthen internal validity. Although we analyzed the transversal parameters, we could not evaluate the sagittal and vertical skeletal dimensions because of the limited field of view, which represents one of the study’s limitations.^7^

Previous researchers have also previously documented age-related and gender-based variations in sinus volume. Aktuna Belgin et al.^19^ and Ariji et al.^20^ demonstrated that sinus volume tends to increase until approximately 20 years of age and subsequently decreases with aging, without significant gender differences. Our mean sample age (22.19 ± 7.71 years) was consistent with this developmental stage. Although the predominance of female patients in our cohort parallels earlier studies, the split-mouth design of our study ensured that age and gender did not influence comparative outcomes.^6-8,21-23^

Previous studies indicated that maxillary sinus morphology may be influenced by tooth loss. Möhlhenrich et al.^24^ found that sinus volume decreased with partial or complete edentulism, whereas Wu et al.^25^ reported a positive correlation between sinus volume and maxillary height, which diminishes after tooth extraction. Therefore, to eliminate potential confounders, we included only patients without missing teeth (except third molars) in the present analysis.

Our results also demonstrated narrower maxillary and molar basal widths on the impacted side, which is consistent with the findings of Tadinada et al.,⁶ Arboleda-Ariza et al.,⁷ and Arriola-Guillén et al.,⁸ who reported smaller transverse and alveolar dimensions in impacted canine cases. These observations underscore the clinical importance of evaluating the transverse dimension in orthodontic planning for such patients. Indeed, previous studies on rapid maxillary expansion reported that transverse widening can lead to a volumetric increase in the maxillary sinus.^26,27^

With regard to nasal morphology, Osman et al.^28^ demonstrated a significant relationship between palatal canine impaction and nasal septum deviation. Accordingly, our study showed a greater septal deviation angle on the impacted side, in which each one-unit increase in deviation corresponded to a 1.177-fold higher likelihood of canine impaction. Conversely, Jongkhum et al.^29^ reported no correlation between septal deviation and maxillary width, which highlights the variability among populations. On the nonimpacted side, a negative correlation between sinus intensity and maxillary transverse parameters suggested that a wider maxilla may be associated with more normalized sinus density.

Although in our study, alveolar pneumatization was slightly greater on the impacted side (51.06%) than on the nonimpacted side (48.94%), the difference was not significant, which is consistent with the literature describing pneumatization as a physiological variation. Nevertheless, to obtain a more objective and reproducible assessment of such subtle anatomical variations, the present study used semiautomatic segmentation via ITK-SNAP software, which, to our knowledge, is the first application of this method for the volumetric and morphometric evaluation of the maxillary sinus in impacted canine cases. We chose ITK-SNAP because its voxel-based active contour algorithm enables anatomically faithful and highly repeatable delineation of soft-tissue and bony boundaries directly from gray-scale intensity differences while also allowing for the automated estimation of density-related parameters derived from voxel intensity information. Although ex vivo tooth studies have reported a slight systematic overestimation of volumes as compared with the water-displacement gold standard, ITK-SNAP remains a widely validated tool for CBCT-based morphometric and density-associated analyses, particularly in anatomically complex structures.^30^

The dimensions of the maxillary sinus might have an influence on various clinical procedures frequently performed in cases with impacted canines, such as the use of miniscrews, orthognathic surgery, and surgical exposure for canine eruption guidance. It should be noted that sinus size may vary depending on age and skeletal maturation; however, because this study used a split-mouth design, each patient served as their own control. This eliminated most of the interindividual variability (bone density, growth potential, gender, etc.). The narrower transverse maxillary dimensions and increased septal deviation angles we observed on the impacted side emphasize the importance of obtaining a detailed 3D assessment before the intervention. Such anatomical variations may influence treatment-planning decisions, for example, including maxillary expansion in orthodontic treatment planning, determining surgical exposure site and the path for eruption, and taking the necessary precautions to prevent potential sinus perforations. Incorporating a CBCT-based morphometric evaluation into routine diagnostic protocols can therefore improve the accuracy of clinical assessments, optimize treatment mechanics, and enhance overall treatment outcomes in complex impaction cases.

This study has several limitations. First, due to the limited field of view of the CBCT images, we could not evaluate the vertical and sagittal skeletal relationships. Second, the study’s retrospective design prevented the control of potential confounding factors, such as sinus pathology or nasal airway variations, at the time of imaging, because no concurrent ear, nose, and throat examination was available. The lack of interexaminer repeatability evaluation is also another limitation. Despite these limitations, this study is the first to employ a semiautomatic segmentation approach using ITK-SNAP 3.8.0 (Cognitica, Philadelphia, PA, USA) to assess maxillary sinus volume and morphology in patients with palatally impacted canines. Future prospective studies with larger and more diverse samples and the integration of 3D volumetric analysis programs are needed to validate and expand these findings.

## Conclusions

No significant differences in maxillary sinus volume were found between the sides with and without impacted upper canines. The molar basal and maxillary widths were significantly narrower on the impacted side.

The increased angle of septal deviation observed on the impacted side suggests potential anatomical asymmetry associated with canine impaction. These findings show that in patients with palatally impacted canines, it is important to evaluate the transverse maxillary dimensions during orthodontic treatment planning and surgical interventions, as these factors could influence space management, miniscrew placement, and surgical access.

## Data Availability

The datasets generated during and/or analyzed during the current study are available from the corresponding author on reasonable request.
